# Evaluating Caregiver-Clinician Communication for Tracheostomy Placement in the Neonatal Intensive Care Unit: A Qualitative Inquiry

**DOI:** 10.21203/rs.3.rs-2869532/v1

**Published:** 2023-05-04

**Authors:** Kylie Bushroe, Kelly Crisp, Mary Politi, Steven Brennan, Ashley Housten

**Affiliations:** 1. Department of Pediatrics, Division of Newborn Medicine, St. Louis Children’s Hospital and Washington University in St. Louis School of Medicine; 2. College of Health and Human Sciences, Northern Illinois University; 3. Department of Surgery, Division of Public Health Sciences, Washington University in St. Louis School of Medicine; 4. Department of Pediatrics, Division of Allergy and Pulmonary Medicine, St. Louis Children’s Hospital and Washington University in St. Louis School of Medicine

## Abstract

**Objective:**

Identify stakeholders’ tracheostomy decision-making information priorities in the Neonatal Intensive Care Unit (NICU).

**Study Design:**

English-speaking caregivers and clinicians who participated in NICU tracheostomy discussions between January 2017 and December 2021 were eligible. They reviewed a pediatric tracheostomy communication guide prior to meeting. Interviews focused on tracheostomy decision-making experiences, communication preferences, and guide perceptions. Interviews were recorded, transcribed, and analyzed using iterative inductive/deductive coding to inform thematic analysis.

**Results:**

Ten caregivers and nine clinicians were interviewed. Caregivers were surprised by the severity of their child’s diagnosis and the intensive home care required, but proceeded with tracheostomy because it was the only chance for survival. All recommended that tracheostomy information be introduced early and in phases. Inadequate communication limited caregivers’ understanding of post-surgical care and discharge requirements. All felt a guide could standardize communication.

**Conclusions:**

Caregivers seek detailed information regarding expectations after tracheostomy placement in the NICU and at home.

## Introduction

In the Neonatal Intensive Care Unit (NICU), about half of premature infants born before 37 weeks estimated gestational age (EGA) will develop bronchopulmonary dysplasia (BPD). When looking at just those babies born extremely premature, at 28 weeks EGA or less, BPD is diagnosed in as high as 78% of infants and rates of both BPD and tracheostomy placement in these infants has been increasing over the last two decades.^1^ Other diagnoses associated with infant tracheostomies include neurologic diagnoses and upper airway or laryngeal structural anomalies.^2^

Tracheostomies can allow infants requiring long-term mechanical ventilation to be safely discharged home. However, in addition to the usual challenges of a NICU admission, these infants face risks and complications associated with the tracheostomy itself.^3,4^ Tracheostomies can also complicate hospital discharge due to requirements for home nursing, care coordination for durable medical equipment, and caregiver training.^5–7^ Tracheostomies are life-changing procedures that can increase caregiver emotional distress. Despite efforts to prepare families for discharge after tracheostomy, caregivers often report feeling overwhelmed, terrified, frustrated, and unprepared upon arrival home.^8,9^

Family-centered communication, education, and engagement on tracheostomy placement can support families through this difficult care transition. We know that caregivers of children in pediatric and cardiac intensive care units seek information about the nature of illness, alternatives to tracheostomy, chance of eventual ventilator-independence, quality of life, potential complications, and expected healthcare needs after discharge.^10–12^ In addition, they desire comprehensive information presented with honesty and transparency from the medical team, and time to process the information in private discussions.^13,14^ Despite this, qualitative research has shown that medical teams often minimize the burdens of tracheostomy to emphasize its benefits.^15,16^

Decision guides are designed to aid in medical decision-making and have been shown to increase patient knowledge, decrease decisional conflict, and improve clinician-patient communication.^17^ Through a literature review, a few tracheostomy decision guides were identified. In particular, we chose a family-centered guide, “Child Tracheostomy: Decision Guide,” created by the Winnipeg Regional Health Authority with caregiver participation to facilitate conversations about tracheostomy placement.^18^ The aims of this study were to identify caregivers’ and clinicians’ informational priorities during the infant tracheostomy decision-making process, assess their perceptions of the family-centered communication guide, and explore their views on implementing a communication guide like it in the NICU.

## Materials and Methods

### Study Design

We conducted semi-structured interviews with clinicians and caregivers to explore their tracheostomy communication and decision-making experiences as well as their perceptions of the example communication guide.

### Human Subjects Protection

The study protocol was approved by the Washington University in St. Louis Institutional Review Board (IRB).

### Participants

Two cohorts of participants were eligible for the study. The first included English-speaking caregivers with primary custody of infants admitted to the NICU who discussed tracheostomy placement for their infants between January 2017 and December 2021. Both those who received and those who declined tracheostomy were eligible. The second cohort included English-speaking clinicians caring for NICU infants who regularly participate in tracheostomy discussions with caregivers. Eligible clinicians included attendings, fellows, and nurse practitioners from the pediatric divisions of neonatology, allergy and pulmonology, otolaryngology, and palliative medicine, and NICU bedside nurses.

Potential caregiver participants were identified by referral from research staff and via electronic medical record search for “tracheostomy” (ICD-10 code Z93.0) in charts of NICU patients admitted between the study inclusion dates. We completed an individual chart review of each patient from the query, and patients were excluded if tracheostomy was discussed after NICU discharge, if the family required an interpreter, or if the caregiver no longer had primary custody. Clinician participants were identified via referral by research staff.

### Data Sources

If participants expressed interest, a teleconference interview was scheduled, and research staff emailed the example guide along with study information and consent letter. Verbal consent was provided at the beginning of each interview. The remainder of the interview was audio-recorded. The IRB-approved interview guide was based on the Consolidated Framework for Implementation Research (CFIR)^19^ and the Ottawa Decision Support Framework.^20^ The interview explored participant informational priorities in the decision-making process, perceptions of the example guide, and views on implementing it in the NICU. Refer to Appendix 1 and 2 for the caregiver and clinician interview guides, respectively. Once the recording ended, participants completed a brief demographic survey. With caregiver consent, research staff collected infant clinical data from electronic medical records including date of birth, gestational age at birth, length of stay in the NICU, and co-morbidities.

### Data Analyses

Audio recordings were professionally transcribed verbatim, and the interviewer reviewed the transcripts prior to analysis for accuracy. Using an inductive/deductive approach, investigators KB and AH, a research team member with expertise in qualitative research methods, developed a codebook based on the interview guide and the Ottawa Decision Support Framework. Standard qualitative analysis procedures were then followed. A minimum of two investigators (KB, AH, KC) independently coded all interviews. The team met regularly to resolve coding discrepancies through consensus, discuss updates to the codebook, and improve understanding of the codebook to achieve consistent application across transcripts. Additional codes identified in the transcripts were added to the codebook iteratively and updates were applied to all transcripts.

Next, the research team met to explore the relationships and concepts within and across transcripts.^21,22^ The research team outlined patterns to identify conceptual linkages and themes. Per standard protocol, recruitment ended when the interviews reached thematic saturation, and no additional themes were identified during analysis.^23^

## Results

Between November 2021 and October 2022, the research team called 35 potential caregiver participants, spoke with 24, and consented 10 (42%) for participation. Concurrently, we emailed 14 potential clinician participants and consented 9 (64%) for participation. Those who declined either were not interested or had scheduling conflicts that prohibited participation.

Participant characteristics are described in Table 1. Of the caregivers, eight identified as female; six were White, three were Black, and one was Asian. All completed at least some college education. All participating caregivers pursued tracheostomies for their infants, and all infants were living at home at the time of interview. Eight infants received tracheostomies for BPD with average age of 148 days (range 100–245 days) at time of tracheostomy. Their corrected gestational ages ranged from 41 weeks and 1 day to 56 weeks and 6 days. The average hospital length of stay was 275 days (range 212–390 days). Seven of the eight were discharged on home ventilators. Two infants received tracheostomies for structural airway anomalies at 5 and 85 days of life. Their corrected gestational ages were 36 weeks and 4 days and 51 weeks and 0 days. Hospital lengths of stay were 46 and 262 days, respectively. One of the two infants was discharged on a home ventilator.

Of the clinician participants, five were physicians, two were nurse practitioners, and two were bedside nurses. They worked in the pediatric divisions of neonatology, allergy and pulmonology, otolaryngology, or palliative medicine. Six identified as female; all were White.

Analysis of the interview transcripts identified four phases in the tracheostomy process: 1) preparation, 2) deliberation, 3) action, and 4) home management and reflection. Definitions of phases and considerations in each are described in Table 2.

### The Preparation Phase

In the preparation phase, providers introduce tracheostomy as an option to caregivers. During this time, clinicians assess caregivers’ emotional readiness for such a discussion. Caregivers described this time as overwhelming and stressful, articulating both surprise and denial that their infants’ diagnoses were severe enough to warrant tracheostomies for discharge. One caregiver explained:

“A lot of the decision-making around placing the trach is being in denial, and that was definitely me. I was totally in denial for how sick <Name> was and what I thought his life should look like… versus what his life is probably going to look like, and once you kind of accept that… it’s hard.”

[Caregiver, P101]

Clinicians echoed this sentiment:

“Sometimes what you say and what they can hear in those moments is different. It doesn’t mean that we didn’t try or that we didn’t provide some of that information. But it… is often stressful and overwhelming … and so I think sometimes … the parents are just at a point where they can’t… absorb all of that.”

[Clinician, P121]

Clinicians also recognized that it can be a shift in caregivers’ thinking and planning:

“It’s a very fundamental change in the way that they will have to care for their child, and it is a very obvious difference from the baby that they probably imagined that they would have and so it can be a hugely emotional conversation.”

[Clinician, P126]

The intensive home care that is required appeared to induce fear amongst caregivers including worries of what living with a child with a tracheostomy would entail, and whether they would be capable of providing the care needed. Caregivers thought that being introduced to the tracheostomy early in the hospital course and then revisiting it multiple times with small amounts of information would help alleviate this stress:

“I don’t know if they’re taught to do it that way or if that’s just kind of how it happens. But… it would kind of come up, but then it would be like, ‘But we don’t need to talk about that now.’ I think that helped that we didn’t try to know the ins and outs of it before we actually had to. But we were hearing it.”

[Caregiver, P105]

The clinicians thought similarly, many thinking the conversations should be started earlier:

“I don’t know if we start those conversations soon enough to help families prepare for the possibility. Saying that this might be a possibility, and then it not being, would be better than not getting them there in the first place.”

[Clinician, P125]

“I think that [medical] decisions happen over a continuum… as opposed to in a moment. And so… I like to think of planting seeds for decisions… that really … are more information guideposts … than asks. With that in mind, you know, at the point in time where the natural history of the disease… has a reasonable chance of leading to tracheostomy, I think that the concept needs to be in front of the family as one of the sets of outcome options, as opposed to now we need to talk about it.”

[Clinician, P129]

Both caregivers and clinicians agreed that the medical team member with the best rapport with the family should take part in the initial conversations.

“I wanted to hear [about tracheostomy] from, you know, somebody that was really following him clinically and would actually be involved in the decision-making process. So, hearing it from the fellows, in our case, was great because they had the time to talk to us.”

[Caregiver, P102]

A caregiver participant suggested:

“I don’t think it would hurt to ask, ‘well [what member] of your medical team do you trust, and who… would you like to hear information from?’”

[Caregiver, P109]

### The Deliberation Phase

In the deliberation phase, caregivers and clinicians consider the information provided, ask questions, and weigh the perceived options. Options may include allowing the patient more time in the NICU, seeking second opinions, proceeding with tracheostomy, or transitioning to comfort care. All caregivers listed similar information they wished they had known during the deliberation phase. This included the steps to discharge home after tracheostomy placement, the process to decannulation after hospital discharge, what living at home with an infant with a tracheostomy would look like, and potential complications. Caregivers made statements such as:

“The resounding thing is it’s so helpful seeing <Name> doing so well and living life like a normal toddler because we didn’t think that was possible after a trach. We thought he was just going to be tied to a ventilator the rest of his life… or, you know, it somehow would affect his mental capacity. So, I think that’s the only other thing that [would be] really helpful, not just the clinical parts of the decision-making process, but what you might be able to expect afterwards.”

[Caregiver, P102]

“I do not feel like the NICU team provided any information like we needed about what was to come after the trach surgery… nobody said anything about… the [possible] bad things after the surgery.”

[Caregiver, P109]

However, during this phase clinicians described that they often focus on the positive aspects and skim over risks and complications of the tracheostomy in fear of upsetting or distancing families:

“When I hear conversations or talk to families about [tracheostomies], we focus on, ‘Oh, it’s great for the baby’s development.’ While it is, it also has this [extra] burden on the family, and I feel like sometimes we’re nervous to talk about negative stuff like that… but, really, it is something that we need to be transparent about. And too… we do all the teaching with families, and then we get them to the point of [being] ready to go home, and then… they’re here, stuck because we can’t find any home nursing.”

[Clinician, P127]

Both caregivers and clinicians recognized that most clinicians could provide limited information about the realities of home life since they don’t have children with tracheostomies at home.

“Providers, doctors, and nurses, I mean, obviously you know about trachs. You know the science; you know how to do the procedure. But the at-home, day-to-day care… no provider can really help you with that, because this is work, and then you go home, and you live your life… unless you have… a child with a trach, which… I haven’t come across a provider that has a child with a trach. So [an online support group] helped a lot in the preparation for going home.”

[Caregiver, P110]

“I don’t know if we fully prepare families for what it’s like… you know, and only because I’ve never experienced it. I’ve only experienced it through what our families have told us.”

[Clinician, P125]

Nearly all caregivers felt that speaking with other families of infants with tracheostomies, whether in-person or online, was the best support when proceeding with tracheostomy. Parents noted:

“Really knowing what it would actually look like for him at home or… afterwards is kind of what was the tipping point of like, ‘Yeah, we’re ready [for a tracheostomy].’”

[Caregiver, P106]

“I think it’s important to show…what a trach baby looks like in everyday life. Like, your baby can still eat if…he or she… doesn’t have an oral airway… he can still go and do normal baby things. It just looks different with a trach.”

[Caregiver, P107]

### The Action Phase

After deliberation, action is taken. Many caregivers were not initially ready to proceed with tracheostomy and desired to give their infant more time to improve:

“Trach and time, those were the only solutions that anybody ever brought up. And when I took [tracheostomy] off the list… every time I met a new person, before we began the conversation I would say, ‘I’m not considering the trach, so don’t bring that up.’”

[Caregiver, P109]

Others asked for second opinions, from either other academic institutions or their referring hospitals:

“I was like, ‘Hey, [our medical team] is talking about traching him,’ and it was really [the referring hospital’s] feedback and thoughts that helped us make that decision [since] we had that rapport with them for so long already… it really helped guide our decision-making.”

[Caregiver, P101]

Many caregivers and clinicians described oscillating between the deliberation and action phases. In the end, most caregiver participants proceeded with tracheostomy because they perceived it was the only option that would lead to infant survival. None of the caregivers in the sample shared that they considered redirecting to comfort care. In fact, some questioned why there needed to be a discussion at all:

“… I remember asking… why are we having a sit-down meeting to ask us, in a conference room with ten doctors, if it’s okay to do a trach. I’m like, ‘Why wouldn’t we do it?’ … I was confused on that, I remember… I didn’t understand why [we] were even having the discussion… It’s just a medical procedure. Let’s move forward… We understood the reasoning for getting the trach. We didn’t understand reasoning why not to get the trach.”

[Caregiver, P104]

### The Home Management and Reflection Phase

In reflecting on the process, nearly every clinician remarked on how the current process, from the timing of tracheostomy introduction to the information provided, is inconsistent across physicians:

“… I think it would be useful to have a… tool. And if nothing else, just to make the messaging consistent that this is all the [information] and it’s not dependent upon… how much time I have or how much experience [I] have or, you know, [it’s not] different… like I said, between child A [and] child B … it’s consistent.”

[Clinician, P121]

“It’s challenging for families because we as a group don’t really do a good job of working them through the decision-making process, [introducing and discussing the tracheostomy] on a consistent basis.”

[Clinician, P129]

While caregivers and clinicians thought a communication guide would be helpful to facilitate conversations, participants identified some potential challenges. First, caregivers perceived the example guide to be long, and both caregivers and clinicians felt it should be tailored to the NICU environment. For example, creating two different documents based on diagnosis of BPD or other congenital anomalies would allow tailoring the material to suit the differences between the two, including reasons for tracheostomy placement, hospital course, and the path towards decannulation. Infants with BPD have lung disease that may improve after years of time and growth while infants with congenital anomalies or structural airway abnormalities may have more complicated courses including multiple airway surgeries without a clear guarantee of decannulation.

“I think that’s another thing, too, is there should be more education for the different… times a trach is needed. You know, is it needed just for BPD? Because they need time for their lungs to grow? Because that has a different outcome than if the baby has a chromosomal abnormality and can’t initiate breaths on their own. And when you’re… looking for research and for outcomes and things like that, it’s all lumped together. You … can’t find, you know, things just on the babies that needed more time to grow.”

[Caregiver, P102]

Another consideration is that clinicians may be under time constraints and leave the guide for families to read independently. However, multiple caregivers and clinicians reiterated that the discussion that accompanies a guide is more important than the guide itself:

“You guys are coming alongside [families] and educating them…There’s going to be a whole lot more to [the tracheostomy discussion] than just the 14 pages [of the guide].”

[Caregiver, P101]

Lastly, caregivers overwhelmingly expressed that the benefits from tracheostomy placement outweighed their fears. They highlighted benefits such as facilitating discharge home, aiding in comfort and development, participating in therapies, reaching developmental milestones, and improving oral feeding. No caregiver expressed regret over the decision for tracheostomy:

“I think one of the things that you can’t grasp in a guide is how much your child is going to be able to, like, thrive, and you can’t grasp that in a guide. You just have to almost do it and then see that they start rolling and they start sitting up and they start walking and they start doing the normal things.”

[Caregiver, P101]

“But for us, I will say, we feel choosing the trach was the best thing we ever did.”

[Caregiver, P104]

“… I think it’s really important if it can be… stressed just how beneficial this can be to the comfort of the baby.”

[Caregiver, P105]

“I think that if there is a way for people to understand that it just-it gives them life.”

[Caregiver, P110]

## Discussion

This project aimed to understand ways to improve communication in the NICU by identifying caregivers’ and clinicians’ tracheostomy information preferences, exploring their perceptions of the example communication guide, and clarifying their views on its use. It met the goals of qualitative research, not to be generalizable as that of quantitative analysis, but rather to provide deep insight into the experiences of its participants. Here we studied the experiences of caregivers and clinicians during the four phases of tracheostomy discussions.

Current literature supports the themes of frustration, fear, and denial that participants expressed in the preparation phase.^8,9^ Medical teams can consider introducing tracheostomy early in the process, providing manageable amounts of information at multiple time points, and revisiting the topic over time. Caregivers mentioned that they would prefer to receive information earlier in the process, even if it is uncertain whether their child will definitively need a tracheostomy, rather than being surprised and unprepared at time of tracheostomy placement. Discussions which include clinicians whom the family trusts may be better received than discussions led by a medical team member without an existing rapport. Identifying trusted individuals can be done by asking families whom they would like to participate in future conversations.

The themes identified during the deliberation phase echo previous literature. Clinicians tend to focus on positive aspects of tracheostomy placement while minimizing the risks and challenges.^15,16^ This conflicts with caregivers’ informational needs and their desire to understand potential risks. Multiple caregivers voiced that they were confused about what was needed for discharge home after tracheostomy placement and were surprised that discharge was not a more immediate step. Discussions regarding requirements for decannulation in the future were generally vague and did not provide the detail sought by caregivers. Providing more realistic and comprehensive information to caregivers about the benefits and possible complications of tracheostomies in the NICU and clearly discussing the expected hospital course after placement can help to prepare families. Clinicians can facilitate in-person or virtual conversations between caregivers and previous NICU families who have been through the tracheostomy process to provide details about home life and lived experiences for which clinicians have limited insight.

During the action phase, all caregiver participants ultimately agreed to tracheostomy for their infants, but for many, it took time to accept this course of treatment. Clinicians can prepare for caregivers to cycle between deliberation and action phases multiple times and be available to answer questions, provide more information, and aid caregivers in working through their fears. None of our caregiver participants mentioned that they considered redirection of care. In contrast to other diagnoses requiring tracheostomies for a stable airway, the infants of families in our study almost all had BPD, a disease with the potential to improve overtime. This may have created a more straightforward decision for caregivers. Families in other studies who declined home ventilation cited concerns about the medical impact of home ventilation, physical and/or communication restrictions their children would face, and unclear contribution to a “clear and meaningful life.”^24^ Like the caregivers interviewed here, those families felt it would be helpful to hear about the experiences of prior families.

Lastly, when reflecting on the process, clinicians noted that every physician approaches the topic of infant tracheostomy differently. Both clinician and caregiver participants felt that a guide could standardize the process and provide a more structured presentation of information across families. The guide used could be tailored to the NICU population, potentially by creating one for infants with BPD and one for infants with other diagnoses. In both cases, caregiver and clinician participants thought it would be useful for the document to include pictures of nurseries with home equipment, links to videos of families completing daily activities with an infant with a tracheostomy and the equipment needed, and stories from other families who have been through the infant tracheostomy process.

### Limitations

There are some notable limitations to this study. We interviewed a small sample of participants. Despite recruitment efforts, caregivers who declined a tracheostomy and those whose infants passed away were not represented in our study. These are important viewpoints that should be explored in future work. In addition, clinician participants represented a subset of clinicians at one institution. Despite the sample limitations, these qualitative data provide rich insight of participants’ experiences navigating this process. Second, the chosen guide was shared with participants prior to their interviews so that they could review and prepare their thoughts. However, this early introduction could have impacted their thoughts on their experiences and preferences. Third, the interview guide did not specifically probe for thoughts on alternative options to tracheostomy placement but should be included in future work.

### Conclusion

Infant tracheostomy placement can be a stressful and emotional experience for caregivers. The process can be improved by introducing the topic of tracheostomy earlier in the process, providing detailed information regarding expectations after tracheostomy placement in the NICU and at home, as well as pictures and videos of infants living at home with tracheostomies. A communication guide could standardize discussions and facilitate steps to discharge. Results from this study can inform efforts to adapt a communication guide for the NICU setting with the aim to improve caregiver-clinician communication during decision-making.
